# Irisin-regulated lncRNAs and their potential regulatory functions in chondrogenic differentiation of human mesenchymal stem cells

**DOI:** 10.1515/med-2024-1073

**Published:** 2024-11-15

**Authors:** Yijie Chen, Wenqi Sha, Yifan Zhang, Wanyi Kou, Liu Yang, Ruixin Guo, Chenyang Li, Junjie Zhao, Zhenghui Wang

**Affiliations:** Department of Otolaryngology-Head and Neck Surgery, The Second Affiliated Hospital of Xi’an Jiaotong University, Shaanxi, 710004, People’s Republic of China; Department of Otolaryngology-Head and Neck Surgery, The Second Affiliated Hospital of Xi’an Jiaotong University, No.157 Xi Wu Road, Xi’an Shaanxi, 710004, People’s Republic of China

**Keywords:** irisin, chondrogenic differentiation, lncRNAs, co-expression, ceRNAs

## Abstract

**Objective:**

Dysregulation of chondrogenic differentiation is associated with osteoarthritis (OA). The myokine irisin is beneficial in OA treatment; yet, the underlying mechanism is not fully understood. Long noncoding RNAs (lncRNAs) act as important regulators of chondrocyte differentiation. This study was conducted to address the role of lncRNAs in mediating irisin-induced chondrocyte differentiation.

**Methods:**

We investigated the irisin-regulated lncRNA profile change in human mesenchymal stem cells (MSCs) using published whole transcriptome sequencing data. We predicted their potential targets and competitive endogenous RNA (ceRNA) prediction and analyzed their molecular functions using functional enrichment analysis.

**Results:**

More differentially expressed lncRNAs (DElncRNAs) were observed in irisin-treated samples. The top irisin-induced lncRNAs were associated with OA or chondrogenic differentiation, including *XIST*, *PAX8-AS1*, *CASC15*, *LINC01618*, and *DLX6-AS1*. The DEGs co-expressed with DElncRNAs were enriched in skeletal system development, extracellular matrix (ECM) organization, cell adhesion, and inflammation associated pathways. Several lncRNAs likely acted as ceRNAs to regulate downstream mRNAs including *ROR2* and *SORBS1* in in OA or chondrogenic differentiation.

**Conclusions:**

We demonstrate the global regulation of lncRNAs by irisin during chondrogenic differentiation of human MSCs. Further study is required to characterize the key irisin-regulated lncRNAs in chondrogenic differentiation

## 1 Introduction

Cartilage injury is a common clinical problem. Due to the weak regeneration potential of chondrocytes, *in vitro* cartilage tissue engineering holds promise in the repair of damaged cartilage [1]. However, the dedifferentiation of chondrocytes is an obstacle to the generation of tissue-engineered cartilage [2]. During *in vitro* culture, chondrocytes can lose their original phenotypes, exhibiting remarkable molecular and morphological changes [3,4]. The dedifferentiation of chondrocytes is also involved in the pathogenesis of osteoarthritis (OA) [5]. The transcription factor SOX9 plays a crucial role in the suppression of chondrocyte dedifferentiation [6,7]. Lentiviral delivery of exogenous SOX9 has been shown to inhibit dedifferentiation and hypertrophy of chondrocytes [8]. A better understanding of the molecular mechanism governing chondrocyte differentiation is of importance in cartilage tissue engineering.

Increasing evidence indicates long noncoding RNAs (lncRNAs) as important regulators of chondrocyte differentiation [9]. lncRNAs are a class of regulatory RNAs implicated in various biological processes including chondrocyte differentiation [10,11,12]. lncRNA *ROCR* deficiency disrupts human mesenchymal stem cell (MSC) chondrogenesis by reducing SOX9 expression in a cis-manner [13]. lncRNA-*MEG3* interacts with the methyltransferase EZH2 to epigenetically inhibit TRIB2 expression, consequently blocking the chondrogenic differentiation of synovium-derived MSCs (SMSCs) [14]. lncRNA-*DANCR* is upregulated by SOX4 to promote the proliferation and chondrogenesis of SMSCs through interaction with and stabilization of myc protein [15]. lncRNAs can also act as competitive endogenous RNAs (ceRNAs) to modulate chondrogenic differentiation, such as *ADAMTS9-AS2* [16] and *DNM3OS* [17].

The myokine irisin shows the ability to enhance chondrocyte differentiation [18,19,20]. It has been documented that irisin stimulates the proliferation and anabolism of human osteoarthritic chondrocytes through inactivation of p38, Akt, JNK, and NF-κB signaling [18]. Irisin affects all stages of cartilage development and ameliorates OA progression by decreasing cartilage degradation and inflammation [19]. Irisin potentiates osteogenic differentiation of bone marrow MSCs through the Wnt/β-catenin signaling pathway [20]. The exposure to irisin leads to proteomic and transcriptomic changes in different cellular contexts [21,22,23]. However, the lncRNA profile change related to irisin-induced chondrogenic differentiation has not been explored.

In this study, we hypothesized that irisin could promote chondrogenic differentiation through the modulation of key lncRNA mediators. To address this, we analyzed the lncRNA profile in irisin-treated human MSCs based on RNA sequencing data reported by a previous study [24]. The dysregulated lncRNAs and their potential targets involved in irisin-induced chondrogenic differentiation were analyzed.

## 2 Materials and methods

### 2.1 Retrieval and process of public data

The publicly available dataset GSE201594 contains RNA sequencing data that reflect the transcriptomic changes in human MSCs after irisin treatment (100 ng/ml) [24]. The dataset was selected in this study to identify key irisin-regulated lncRNAs in chondrogenic differentiation. Three repeated samples were used for each group [25]. The human MSCs were obtained from healthy volunteer donors. The SRA run files of RNA-seq samples were converted to fastq format with NCBI SRA Tool fastq-dump (v.2.8.0). The raw reads were trimmed to remove ambiguous reads (with N bases) using a FASTX-Toolkit (v.0.0.13; http://hannonlab.cshl.edu/fastx_toolkit/) and then filtered by removing the ending low-quality bases (quality score <20) and low-quality reads (30% bases with quality score <20). The quality of filtered reads was finally evaluated using the FastQC software (http://www.bioinformatics.babraham.ac.uk/projects/fastqc).

### 2.2 Reads alignment and differentially expressed genes (DEGs) analysis

The quality-filtered reads were aligned onto the human GRCH38 genome by HISAT2 (v.2.2.1) with no more than four mismatches. Uniquely aligned reads were screened for further analysis. The expression levels of genes were evaluated by fragments per kilobase of exon per million fragments mapped (FPKM) [26]. The DESeq2 (v.1.30.1) software [27] was used to identify DEGs with fold change (FC) ≥2 or ≤0.5 and *p*-value ≤0.01. We used a false discovery rate (FDR) <0.1 as a threshold value and obtained similar results. RNA-binding proteins (RBPs) on the list of 2,141 RBPs retrieved from previous reports [28,29,30,31] were filtered out from the DEGs.

### 2.3 lncRNA prediction

We combined the aligned results of RNA-seq data to assemble and predict novel transcripts using StringTie2 (v2.1.6) [32]. We eliminated the low abundant transcripts with FPKM <1. To identify lncRNAs from the assembled transcripts, we used 4 tools to predict the coding potential of transcripts: CPC2 (v2.0) [33], LGC (v1.0) [34], CNCI (v2.0) [35], and CPAT (v3.4.0) [36]. The noncoding transcripts longer than 200 bp were picked up as lncRNAs. Differentially expressed lncRNAs (DElncRNAs) were analyzed with the DESeq2 (v.1.30.1) software.

### 2.4 Correlation analysis

To analyze the potential regulatory functions of lncRNAs, correlation analysis was performed between all DElncRNAs and all DEGs. Pearson’s correlation coefficients (PCCs) between DElncRNAs and DEGs were calculated, and lncRNA-target relationship pairs satisfying the absolute value of PCC ≥0.6 and *p*-value ≤0.01 were identified.

### 2.5 Predict the relationship between DElncRNAs and microRNAs (miRNAs)

DElncRNAs can play a role as a sponge of miRNAs [37]. Here, we used two methods to predict the target relationship between miRNAs and DElncRNAs. One method is Miranda (https://anaconda.org/bioconda/miranda, v3.3) that predicts the miRNA complementary pairing with DElncRNA with a Miranda score >150. Another method is rnahybrid (https://bibiserv.cebitec.uni-bielefeld.de/rnahybrid/) that assumes miRNA–DElncRNA pairing with *p*-value ≤0.05. We took the overlapped pairs of the two methods as the final miRNA–DElncRNA target relationship. Finally, we analyzed the miRwalk database (http://mirwalk.umm.uni-heidelberg.de/) to find the differential targeting genes with default parameters.

### 2.6 Functional enrichment analysis

To explore the functions of identified gene sets, the enriched Gene Ontology (GO) and KEGG pathways for a given gene set were identified using KOBAS 2.0 [38]. We downloaded the KOBAS 2.0 pipeline to our local server and used the latest GO and KEGG databases to perform this analysis. Hypergeometric test and Benjamini–Hochberg FDR controlling procedure were used to define the enriched *p*-value and FDR of each pathway.

### 2.7 Statistical analysis

Principal component analysis (PCA) was performed by the R package factoextra (https://cloud.r-project.org/package=factoextra) to demonstrate the clustering of samples with the top two principal components. Unpaired Student’s *t*-test was used to analyze the statistical significance of two samples.

## 3 Results

### 3.1 lncRNAs are globally regulated by irisin in human MSCs

**Figure 1:**
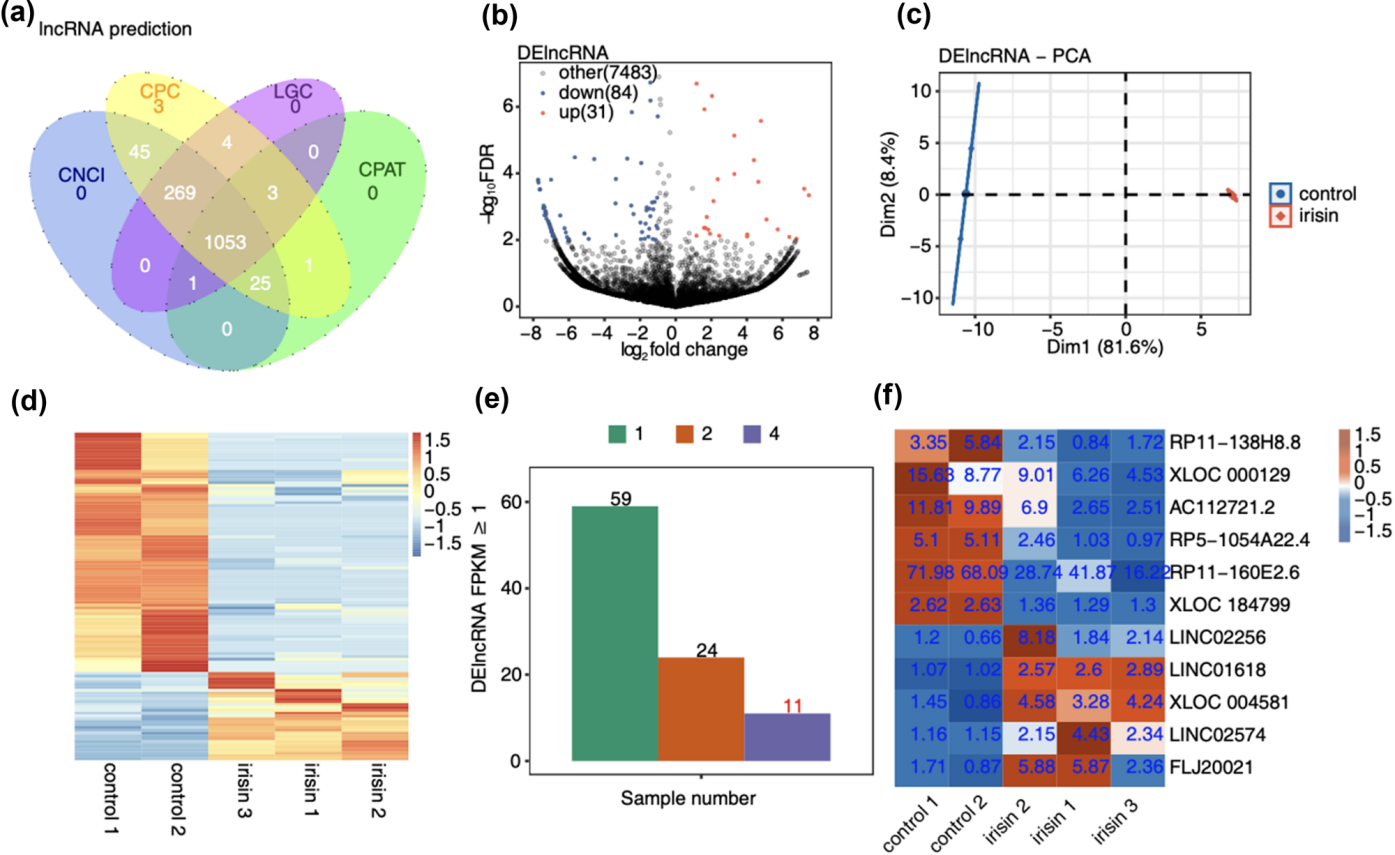
Transcriptional analysis of differential expression lncRNAs in irisin-treated and control samples. (a) The overlap of predicted lncRNAs by the CPC, CNCI, CPAT, and LGC tools. (b) Volcano plot showing all DElncRNA between irisin and control samples with DESeq2. (c) PCA based on FPKM value of all DElncRNA. The ellipse for each group is the confidence ellipse. (d) The heatmap showing the expression level of DElncRNA. (e) The bar graph showing the number of DElncRNA FPKM ≥1, the abscissa is the number of samples, and the ordinate FPKM ≥1. (f) The heatmap diagram showing that the FPKM of DELncRNA of at least 4 samples is ≥1.

**Figure A1:**
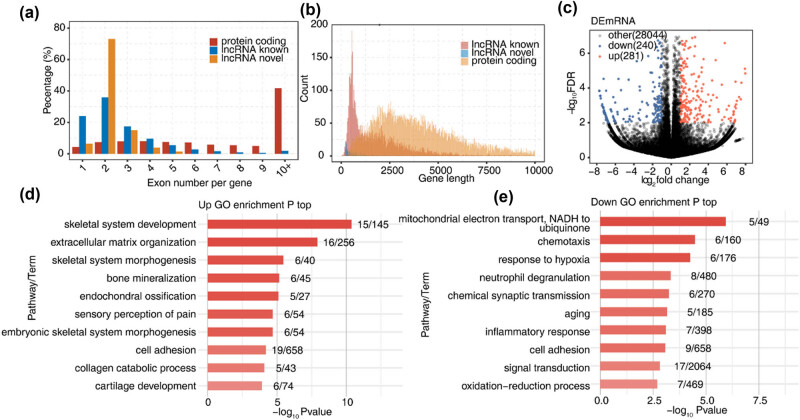
Transcriptional analysis of differential expression lncRNA in irisin and control. (a) Distribution of exon count of known lncRNA, novel lncRNA, and protein coding RNA. (b) Density of the length distribution of known lncRNA, novel lncRNA, and protein coding RNA. The length density distribution was generated by density function. (c) Volcano plot showing all DEmRNA between irisin and control samples with DESeq2. *P* value < 0.01 and FC (fold change) ≥ 2 or ≤ 0.5. (d) Bar plot exhibiting the most enriched GO biological process results of the up-regulated DEGs. (e) Bar plot exhibiting the most enriched GO biological process results of the down-regulated DEGs.

**Figure A2:**
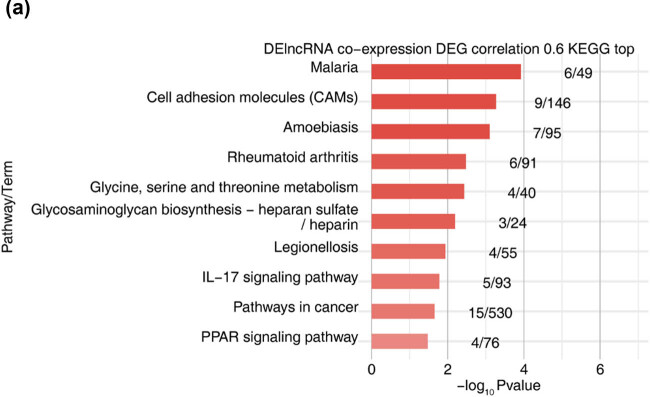
Analysis of interaction between DElncRNA and DEmRNA in irisin and control. (a) Bar plot showing the top 10 enriched KEGG pathways of DEmRNA co expressed by DELncRNA.

We downloaded the transcriptome sequencing data generated using human MSCs treated with irisin, which plays essential roles in glucose homeostasis [39] and chondrocyte biology [40]. The GSE201594 dataset containing three control and three irisin-treated samples [24] was analyzed. The published study using this dataset mainly analyzed the differentially expressed mRNA genes [24]. Here, we focused on the differentially expressed lncRNAs. There was a high overlap among the four methods that were used to predict lncRNAs (Figure 1a). The predicted lncRNAs had fewer exon number than mRNAs (Figure A1a). Transcript length distribution also revealed a shorter length for novel lncRNAs (Figure A2b).

We then performed DEG analysis of both mRNAs and lncRNAs between irisin and control. The down DEmRNAs (240) were similar to the up DEmRNAs (281) (Figure A1c). DElncRNAs analysis indicated 31 up DElncRNAs and 84 down DElncRNAs (Figure 1b and Table A1). PCA revealed the clear separation between irisin and control samples for DElncRNAs (Figure 1c). The hierarchical clustering heatmap also showed the consistently dysregulated expression level for DElncRNAs in irisin and control groups (Figure 1d). Most lncRNAs were lowly expressed in irisin-treated samples (Figure 1e). We then selected the top six down DElncRNAs and five up DElncRNAs for further analysis, including *FLJ20021*, *LINC02574*, *LINC01618*, *RP11-160E2.6*, *AC112721.2*, and *RP11-138H8.8* (Figure 1f). The DEmRNAs were highly enriched in pathways associated with irisin functions (Figure A1d–e).

### 3.2 DElncRNAs are potential regulators for DEmRNAs in response to irisin

**Figure 2:**
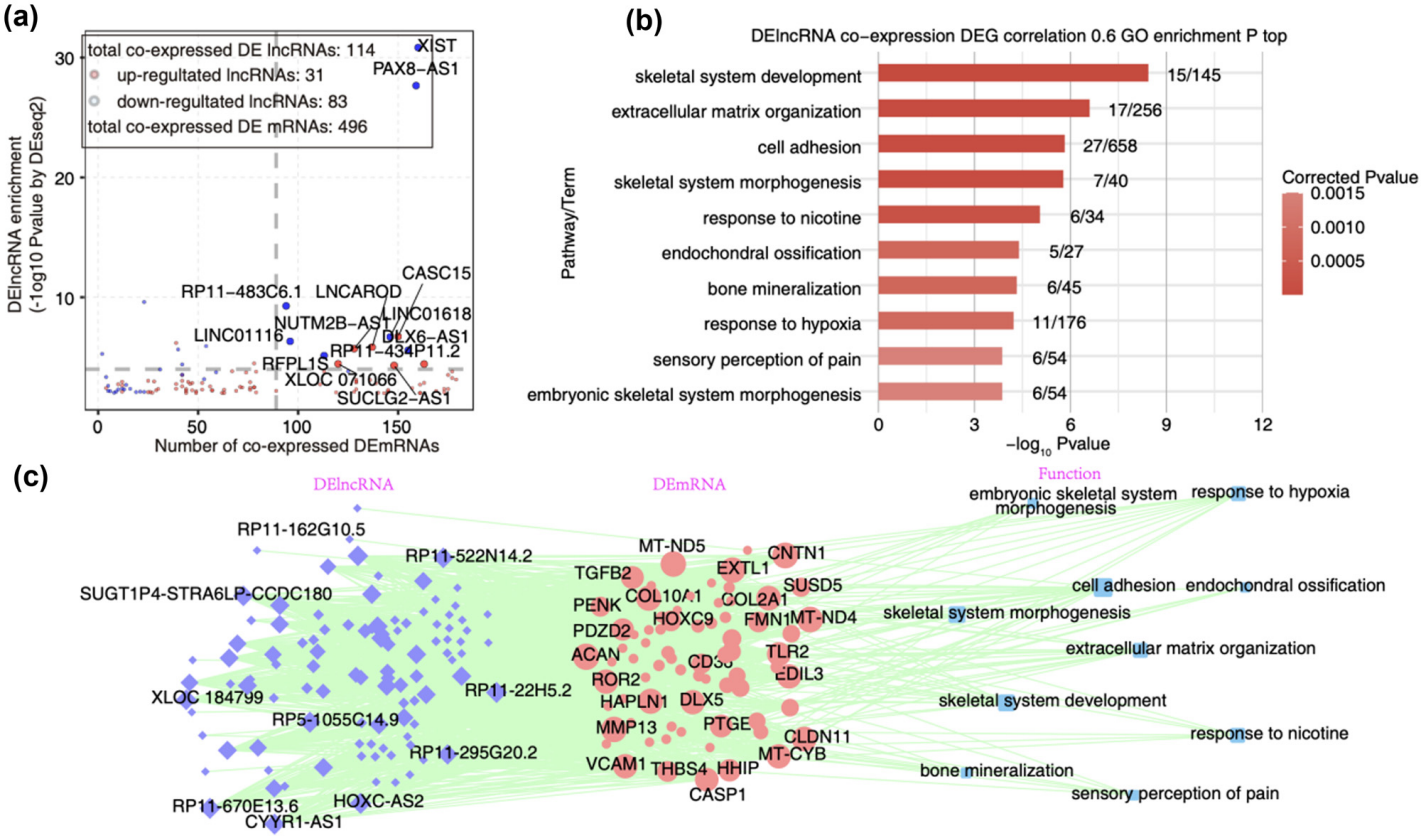
Analysis of interaction between DElncRNAs and DEmRNAs in irisin vs control samples. (a) Scatter plot showing DElncRNAs by irisin compared with control samples and its number of co-expressed DEmRNAs. Red points denote up-regulated lncRNAs involved in co-expression pairs and blue points denote down-regulated lncRNAs. Cutoffs of *p* value <0.01 and Pearson coefficient >0.6 were applied to identify the co-expression pairs. (b) Bar plot exhibited the most enriched GO biological process results of DEmRNAs co-expressed with DELncRNAs. (c) Network diagram showing top 10 GO biological process results of DEmRNAs regulated by DElncRNAs.

As regulatory RNAs, lncRNAs have ability to modulate gene expression in cis- or trans-manners. Co-expression analysis is a canonical method to explore the regulatory pairs between regulators and their targets [41]. We thus analyzed the significant correlation pairs between DElncRNAs and DEmRNAs (see details in materials and methods). The DElncRNAs detected were co-expressed with 496 DEmRNAs. In particular, the five lncRNAs were significantly correlated with more DEmRNAs, including *XIST*, *PAX8-AS1*, *CASC15*, *LINC01618*, and *DLX6-AS1* (Figure 2a). We then explored the enriched functions of co-expressed DEmRNAs using GO and KEGG databases. The top 10 enriched GO biological processes contained skeletal system development, extracellular matrix (ECM) organization, cell adhesion, skeletal system morphogenesis, and response to nicotine (Figure 2b). Meanwhile, endochondral ossification and bone mineralization were also enriched (Figure 2b). The KEGG pathway analysis revealed several enriched diseases and pathways, including rheumatoid arthritis (Figure A2a). To better display the co-expression pairs between DElncRNAs and DEmRNAs, we performed network presentation for DElncRNAs, DEmRNAs, and their enriched GO BP pathways to demonstrate the co-expression network and the co-expressed strength (Figure 2c). These results indicate that DElncRNAs have the potential to broadly regulate the expression of DEmRNAs in response to irisin in human MSCs.

### 3.3 DElncRNAs act as ceRNAs to regulate expression of RBPs

**Figure 3:**
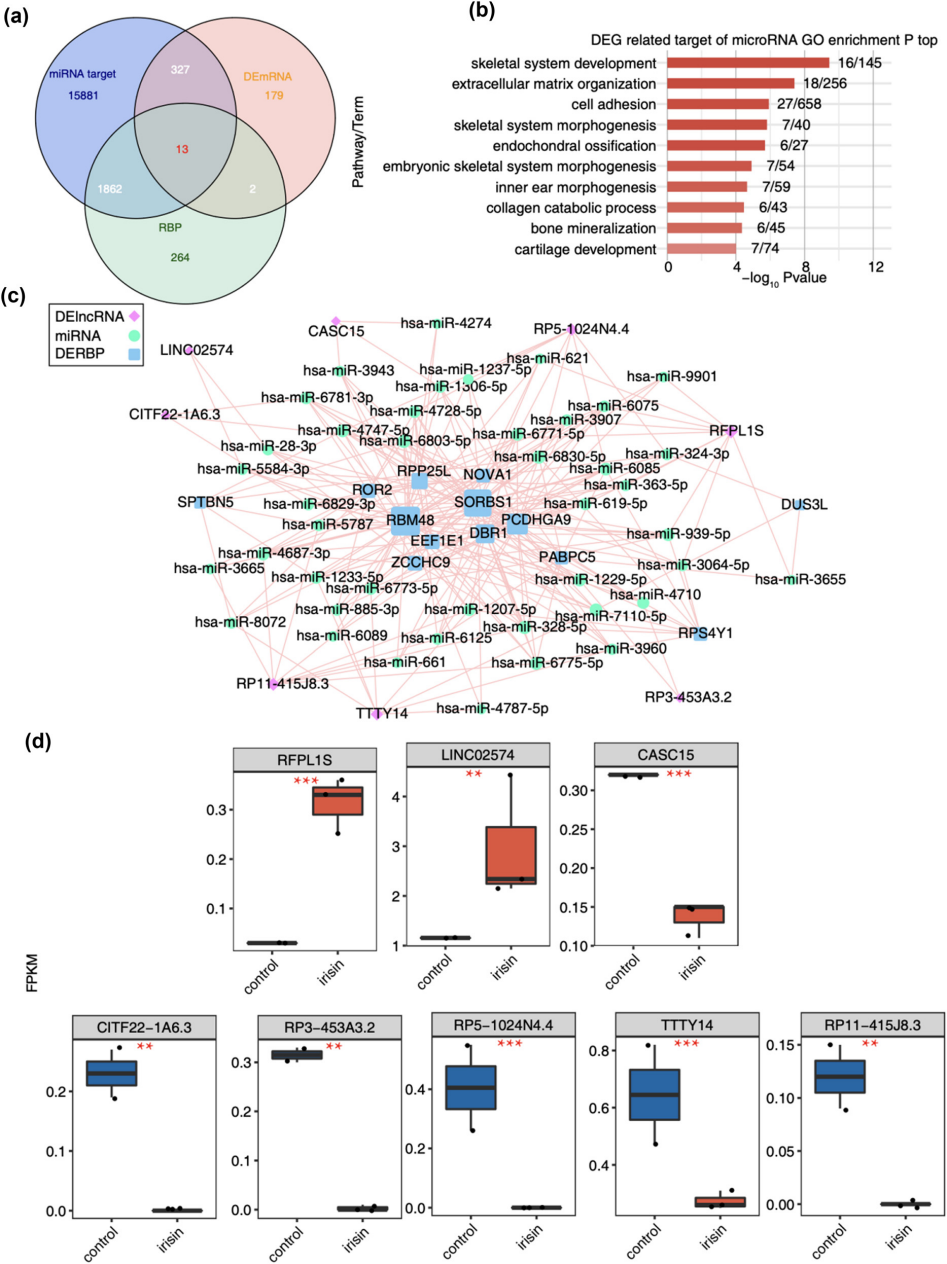
Co-expression network of DElncRNAs–miRNAs–DERBPs between irisin vs control. (a) Venn diagram showing the overlap of microRNA-differential target genes and RBPs. (b) Bar plot showing the most enriched GO biological processes for differential miRNA target genes. (c) Network diagram showing the DElncRNA–microRNA–DERBPs. (d) Boxplot showing FPKM of eight DElncRNA. *p  <  0.05, **p  <  0.01, ***p  <  0.001.

**Figure A3:**
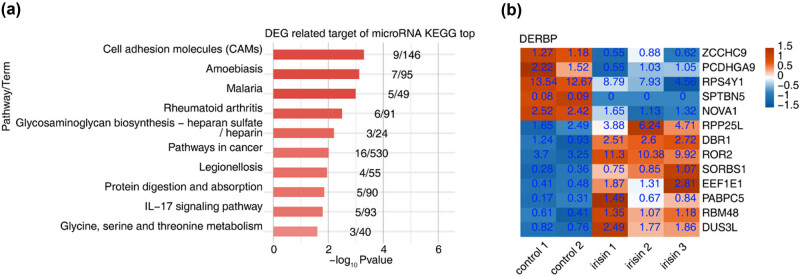
Co-expression network of DElncRNA-microRNA-DERBPs in irisin and control. (a) Bar plot showing the top 10 enriched KEGG pathways of differential miRNA target genes. (b) The heatmap diagram showing the expression profile of DERBPs in Figure 3A.

One canonical functional manner for lncRNAs is to act as ceRNAs with miRNAs, thus indirectly modulating mRNA expression [42]. After predicting the targets of miRNAs using base-pair methods, we overlapped the miRNA targets and DEmRNAs, and identified 340 overlapped mRNAs, occupying 65% of all DEmRNAs (Figure 3a). Functional enrichment analysis of these overlapped DEmRNAs revealed similar enriched pathways with all DEmRNAs (Figure 3b and Figure A3a), suggesting that DEmRNAs could be broadly regulated by miRNAs. As one large family of proteins, RBPs play essential roles in transcriptional and post-transcriptional regulation [30]. We found that 13 out of 15 overlapped RBPs were miRNA targets (Figure 3a), indicating that RBPs were more likely to be regulated by miRNAs. Among the 13 RBPs, 8 were up-regulated and 5 down-regulated (Figure A3b). We then plotted the regulatory network for DElncRNAs–miRNAs–DERBPs. Several DERBPs, including *RBM48*, *SORBS1*, *PCDHGA9*, *RPP25L*, and *ROR2*, were highly regulated by more miRNAs than other DERBPs (Figure 3c). Eight DElncRNAs were involved in this regulatory network, two up-regulated and six down-regulated (Figure 3d).

## 4 Discussion

Dedifferentiation or degradation of cartilage leads to multiple diseases including OA [3]. Identification of the key factors that can prevent chondrocyte dedifferentiation is of significance in cartilage repair. Irisin plays an important role in a wide range of diseases such as OA, sarcopenia, metabolic diseases, and neurological diseases [43,44]. *In vitro* studies have indicated irisin as a positive regulator of chondrogenic differentiation [18,19,20]. In this study, we demonstrate the alteration of lncRNA expression profile by irisin in human MSCs and describe the potential lncRNAs and downstream targets involved in chondrogenic differentiation. Our work sheds light on the regulation of lncRNA mediators by irisin and reveals novel regulatory lncRNAs during chondrogenic differentiation.

Previous studies have demonstrated that irisin treatment elicits proteomic and transcriptomic changes in multiple types of cells [21,22,23]. For instance, Dehghan et al. [22] reported that irisin injection alters dozens of proteins in murine brain cells. Mathias et al. [23] demonstrated that irisin treatment modulates a lot of genes related to the purinergic signaling in differentiated human adipocytes. Consistently, our study indicates that irisin treatment causes a global alteration of lncRNAs in human MSCs. Among the irisin-regulated lncRNAs, several ones exhibit the capacity to modulate the progression of malignant diseases. For instance, the lncRNA *CASC15* promotes the proliferation and invasion of lung cancer cells *via* the miR-766-5p/KLK12 axis [45]. Another lncRNA, *PAX8-AS1*, can inhibit the proliferation and induce apoptosis in papillary thyroid carcinoma cells [46]. However, few lncRNAs have been investigated in the course of chondrogenic differentiation. Intriguingly, we identified two irisin-regulated lncRNAs, i.e., *XIST* and *DLX6-AS1*, which can regulate osteogenic differentiation [47,48]. Liu et al. [47] reported that *DLX6-AS1* is upregulated in dental pulp cells by BMP9 and promotes osteogenic differentiation. Zheng et al. [48] reported that knockdown of *XIST* blocks the osteogenic differentiation of human bone marrow MSCs. Moreover, *XIST* can modulate the proliferation and survival of chondrocytes and thus contribute to the progression of OA [49,50]. These studies, combined with our findings, suggest the possibility that irisin may promote chondrogenic differentiation through modulation of key lncRNAs including *XIST* and *DLX6-AS1*.

Functional enrichment analysis reveals that the irisin-regulated lncRNAs are associated with target mRNAs involved in ECM organization, cell adhesion, bone mineralization, response to hypoxia, and inflammation. Hypoxia-inducible factor 1α regulates chondrogenesis by transactivating the expression of *SOX9* [51]. The lncRNA *XIST* can promote SOX9 expression through the IL-6/STAT3 signaling pathway [52]. In addition, chondrogenic differentiation is regulated by specific ECM components [53]. Notably, *XIST* shows the ability to promote ECM degradation [54]. These findings suggest that irisin-regulated lncRNAs may modulate key transcription factors such as SOX9 to affect ECM deposition and chondrogenic differentiation.

In addition to the direct modulation of target genes, lncRNAs act as ceRNAs to regulate gene expression *via* an indirect manner. RBPs have been reported to regulate anabolic and catabolic gene expression in chondrocytes [55]. In this study, we identified seven lncRNAs that can regulate RBPs by sponging miRNAs. The lncRNAs *LOC100126784* and *POM121L9P* are the upstream ceRNAs of miR-503-5p and promote osteogenic differentiation of bone MSCs by regulating SORBS1 expression [56]. These studies provide another possible mechanism by which lncRNAs regulate chondrogenic differentiation induced by irisin.

Several limitations of this study should be noted. First, the functions of the irisin-regulated lncRNAs in chondrogenic differentiation have not been determined. The lncRNA *XIST* is a promising one mediating the activity of irisin in chondrogenic differentiation. However, there is a lack of direct evidence for this speculation. Second, the regulation of lncRNA expression profile by irisin needs to be validated in clinical samples.

In summary, irisin treatment leads to a global alteration of lncRNAs in human MSCs, which modulate a large number of target genes through direct or indirect mechanisms. Irisin-regulated lncRNAs may serve as potential therapeutic targets in the prevention and treatment of cartilage injury. Further work should be conducted to fully characterize the key irisin-regulated lncRNAs in chondrogenic differentiation.

## Supplementary Material

Supplementary Table
